# Work-Related Health Literacy: A Scoping Review to Clarify the Concept

**DOI:** 10.3390/ijerph18199945

**Published:** 2021-09-22

**Authors:** Anna T. Ehmann, Eylem Ög, Monika A. Rieger, Achim Siegel

**Affiliations:** Institute of Occupational and Social Medicine and Health Services Research, University Hospital Tübingen, Wilhelmstr. 27, 72074 Tübingen, Germany; anna.ehmann@med.uni-tuebingen.de (A.T.E.); Eylem.Oeg@med.uni-tuebingen.de (E.Ö.); monika.rieger@med.uni-tuebingen.de (M.A.R.)

**Keywords:** health literacy, employees, work ability, employability, workplace setting

## Abstract

The overall aim of this scoping review is to outline the current state of research on health literacy (HL) in the workplace: the primary objective is to clarify the concept of individual work-related HL; the secondary aims are to report on interventions that promote individual work-related HL and to present respective measurement instruments validated to date. A high level of work-related HL could support work ability and in the longer run employability. These topics are becoming increasingly important in current circumstances and in view of ongoing developments (e.g., digitalization and “new” work). A basic understanding and measurement of HL as an individual competence in the context of working life is necessary to develop future interventions to promote HL among people of working age. According to the participants, concept, and context (PCC) framework, we included articles on health literacy (concept) in the target group of people of working age in the workplace (population and context). Key information sources were the databases PubMed, CINAHL, PsycInfo, and PSYNDEX. A total of 30 articles were included. There are several terms for “health literacy in the workplace” (including individual work-related or occupational HL). The conceptualizations of the individual employee’s competence covered all aspects of HL (“access”, “understand”, “appraise”, and “apply” health information). The conceptualizations differed, among others, in the covered time horizon (referring either only to employees’ current work situation or additionally to their employability in the lifespan) or whether they referred also to the viability of the respective company. Published interventions attempting to promote individual work-related HL seem mostly to be targeted at the promotion of mental HL. A variety of outcomes have been measured in intervention studies, while specific measurement instruments for individual work-related HL seem to be scarce. We recommend the development of country-specific instruments for the assessment of individual work-related health literacy and to measure mental and physical work-related health literacy.

## 1. Introduction

Individuals’ participation in the workforce plays a central role in providing them a livelihood and a basis for social insurances and social participation. Work is an influential factor during the life course and has a potentially large impact on one’s health and well-being [1]. At work, increasing complexity, rapid changes, and the altered working environment as a result of globalization and digitalization are causing—amongst other things—an increase in self-employed people and the blurring of work–life and other areas of life [2,3,4]. As a consequence, state-imposed occupational health and safety measures that are established by companies at workplaces reach employees even less well during their “new” or mobile work [2,3,4,5], since even the implementation of legally prescribed measures for occupational health and safety in workplaces can be deficient [6]. In consideration of changes in work environments, including tendencies toward greater flexibility and altered employment biographies, managing one’s own health is increasingly gaining in importance [7,8]. In Germany, with its 83 million inhabitants, there was a workforce of more than 46 million people in 2019 and 2020 [9].

### 1.1. Health Literacy

Enhanced individual health literacy of employees can enable them to independently shape working conditions (hazards as well as protective measures) as a behavioral preventive measure and, thus, contribute to the implementation of structural preventive measures [10]. From the original use of the term “health literacy” in the 1970s in the context of education [11], the numerous publications and ongoing research in the field of public health demonstrate the increasing importance and relevance of health literacy. A comprehensive definition or conceptualization of health literacy synthesizing previous literature was developed by Sørensen et al. [12]. Thus, health literacy “entails people’s knowledge, motivation and competences to access, understand, appraise, and apply health information in order to make judgments and take decisions in everyday life concerning healthcare, disease prevention and health promotion to maintain or improve quality of life during the life course” [12].

### 1.2. Health Literacy in the Context of Employment

Individuals’ health-related decisions based on relevant information can also be beneficial in the context of work and health, such as in questions regarding recovery after illness, sickness absence, and rehabilitation [13] back into community and working life. Limited health literacy among employees can also be one of the factors limiting the understanding and training effectiveness of occupational health and safety [14]. In occupational settings, health literacy can be allocated to these two components of workplace health management in Germany: “occupational health and safety” as well as “workplace health promotion” [4]; however, especially with regard to longer working lives and the blurring of work and other areas of life, employees’ own contributions to maintaining their work ability are becoming increasingly important in order to ensure that the living and working conditions of employees are safe and healthy [4]. Work ability is defined as the potential of the individual to fulfil his or her work tasks, considering personal health, working conditions as well as mental resources [15,16]. Work ability comprises individual as well as work-related factors [17]. Given that these various factors (such as health problems or physical and psychological demands of the work) change with age, work ability is determined by the work environment, the contents of the work, and social relationships as part of the work as well as by the individual factors of the employee [17].

### 1.3. Relevance of Health Literacy in the Context of Employment and Current Importance

Additionally, in consideration of the workforce’s aging, employees’ health literacy is of growing importance [4]. In order to maintain overall productivity and competitiveness, enterprises are required to develop measures to promote the work ability of their employees [18,19]. However, young employees’ health literacy is also of growing importance [20]. Stassen et al. demonstrated the significant influence of health literacy domains on work ability in young employees [20].

The promotion of health literacy, and especially of health literacy addressing knowledge and skills in occupational settings, can lead to improved individual skills, less occupational hazards and injuries, and to the maintenance and promotion of work ability. In the past five years, interdisciplinary collaborations in health literacy research evolved between, for example, environmental or occupational health, public health, and nursing [21,22,23,24].

At the beginning of 2020, the disease COVID-19, caused by the coronavirus SARS-CoV-2, developed into a pandemic [25]. Mobile work, as work that is not performed at a workplace, but where professionals work at any other location (e.g., their home) [26], increased in the course of the COVID-19 pandemic as measure for the infection protection [27]. These current circumstances may lead to a greater relevance of the individual employees’ awareness and ability to contribute to staying safe and healthy at work. Work-related health literacy may contribute considerably to this.

### 1.4. Operational Definition of Work-Related Health Literacy and Scoping Review Objective

Our operational definition of work-related health literacy refers to employees’ knowledge of one’s own work-related risks, threats, and requirements. This includes knowledge of one’s own working conditions, needs, and knowing about available support inside and outside the workplace. An example for “support outside the workplace” is the awareness of social insurance offers that are available for one’s own. As we consider work-related health literacy to be an individual competence, we excluded articles on organizational health literacy that conceptualized “health literacy” as a property of organizations and not of individuals. The objective of this review is to provide an overview of the current state of research on individual health literacy in the workplace and to contribute to clarifying the concept of work-related health literacy. This goal can be achieved very well with a scoping review [28]. Since scoping reviews have a wide range of uses [29,30,31] and aim to provide an overview on a topic or to develop a mapping of the literature [29,31,32], we opted for this method.

## 2. Methods

First of all, we conducted a preliminary literature search. Based on a first search in the databases of Joanna Briggs Institute (Systematic Review Register) [22] and the Cochrane Library [23], there were no current or ongoing reviews on the topic, and the database search for measurement instruments (Health Literacy Tool Shed [24]) measuring health literacy in the workplace revealed no results, too.

### 2.1. Review Question

To elaborate the review question, we used the PCC (participants, concept, and context) framework [32]: focusing on employees in the workplace setting, we combined participants and context, while “health literacy” was our relevant concept.

The main research question (RQ 1) is: How is individual work-related health literacy defined or conceptualized?

In addition, we answer the following two questions:

- RQ 2: What measures or interventions are available to promote work-related health literacy in individual employees?
- RQ 3: How can individual work-related health literacy be measured?

### 2.2. Eligibility Criteria

#### 2.2.1. Participants and Context

Articles on individual work-related health literacy or—as a synonym—occupational health literacy in the target group of employed people of working age were included. The International Labour Organization (ILO) defines people of working age usually as persons at the age of 15 and over [33]. No particular professional groups were excluded.

#### 2.2.2. Concept

We included articles that contributed to the conceptualization of individual work-related health literacy, reported on interventions to promote individual work-related health literacy, or presented measurement instruments for individual work-related health literacy. Articles lacking work-relatedness, i.e., missing a link to the maintenance of work ability or employability, were excluded. Articles that focused on promoting health in general (e.g., nutrition or exercise programs) and only made use of the workplace setting (e.g., to easily recruit study participants) were excluded.

#### 2.2.3. Types of Sources

RQ 1: For the first research question, this scoping review considered all types of articles, e.g., case studies, qualitative studies, theoretical articles, and reports. Chapters of books were also included, while individual opinion papers, brochures, research programs, announcements, articles in newspapers, and congress proceedings such as abstracts or presentations were excluded.

RQ 2: For the second research question, intervention studies were included. In the iterative process of the scoping review in gathering measures of work-related health literacy, we only included studies providing quantitative outcomes after the implementation of the measures. Thus, for RQ 2, we omitted process evaluations or impressions on the implementation of measures. Interventions aiming to reduce the sick days of employees—without surveying aspects of work-related health literacy—were not included. For the presentation of intervention studies, study protocols and articles with exclusively descriptive contents were excluded.

RQ 3: For the third research question, we considered studies that reported on the development and validation of instruments that survey individual work-related health literacy. Instruments for healthcare professionals aiming at improving patient care were excluded from this scoping review, as well as instruments that predominantly asked about knowledge of how to recognize health problems in colleagues.

This scoping review was registered on Open Science Framework (https://osf.io/8dsmf, registered on 10 September 2020, accessed on 20 Septmeber 2021) [34] and is conducted in accordance with the Joanna Briggs Institute (JBI) methodology for scoping reviews [32]. We used the preferred reporting items for systematic reviews and meta-analyses extension for scoping reviews (PRISMA-ScR) [30] (see Supplementary Materials Table S1). The a priori protocol of this scoping review has not been published.

### 2.3. Search Strategy

The search strategy aimed to locate both published and unpublished articles. An initial limited search of PubMed and SpringerLink was undertaken to identify the first articles on the topic. The text words contained in titles and abstracts of relevant articles as well as index terms to describe the articles were used to develop a full search strategy for PubMed. The creation and editing of the search string for PubMed was done in exchange with the Medical Library of Tuebingen (D.M., see acknowledgements section). Furthermore, feedback was provided according to the PRESS Guideline [35] by a health services researcher with experience in conducting reviews (A.W.) [36,37,38]. The developed PubMed search string was adapted to each of the other databases CINAHL, PsycInfo, and PSYNDEX (see Appendix A). Another search strategy was implemented for the hand search (German language). Sources of unpublished studies/gray literature included articles of the Joint German Occupational Safety and Health Strategy (Gemeinsame Deutsche Arbeitsschutzstrategie, GDA) [39], the Federal Institute for Occupational Safety and Health (Bundesanstalt für Arbeitsschutz und Arbeitsmedizin, BAuA) [40], and SpringerLink. For information sources and search strategy, see Appendix A. Articles published in English or German were included, and there was no restriction on the publication date. The databases to be searched included PubMed as well as CINAHL, PsycInfo, and PSYNDEX via the EBSCOhost interface. All of these last three databases were searched separately via the EBSCOhost interface. Moreover, the reference lists of all included articles were screened for additional sources of evidence.

### 2.4. Source of Evidence Selection

Following the search, in August 2020, all identified citations were collected and uploaded into CITAVI (Swiss Academic Software GmbH, Version 6.3/2020, Switzerland), and duplicates were removed. After a pilot test, titles and abstracts were screened by two reviewers for assessment against the inclusion criteria for the review. Using the software Rayyan (Qatar Computing Research Institute (Data Analytics), Doha, Qatar) [41], the titles and abstracts were screened independently by the two reviewers (A.T.E., E.O.). Any disagreements that arose between the reviewers at each stage of the selection process were resolved through discussion and, in some cases, the involvement of a third scientist (A.S.). Potentially relevant sources were retrieved in full. The full text of selected citations was assessed in detail. Reasons for the exclusion of sources of evidence at the stage of full-text analysis were recorded. To include newly added articles as well, the search was rerun in the specified sources on 14 January 2021.

### 2.5. Data Extraction

Data of included articles were extracted by the two reviewers (A.T.E., E.O.) using a data extraction tool developed by the reviewers. We started the data extraction independently, adapted the tools for each research question, and then, jointly discussed the completed tools. The data extracted included specific details about the study participants and context, the conceptualization of “health literacy in the workplace”, type of article, study methods, and key findings relevant to the research questions. The extraction tool was modified during the screening process and adjusted to the needs of both reviewers.

### 2.6. Data Analysis and Presentation

The results of the three research questions are presented below. First, we present the results on definitions of “health literacy in the workplace” in the form of a table (Table 1). We show the characteristics of further conceptional articles in a “patterning chart” [42] (Table 2). One ingredient of the patterning chart of Table 2 was the four aspects “access”, “understand”, “appraise”, and “apply” health information of Sørensen et al.’s conceptualization of health literacy [12]. The complete frame for Table 2 was developed by the authors themselves on the basis of the included literature.

Results for the other two research questions follow in two further tables (Table 3 and Table 4).

## 3. Results

**Figure 1:**
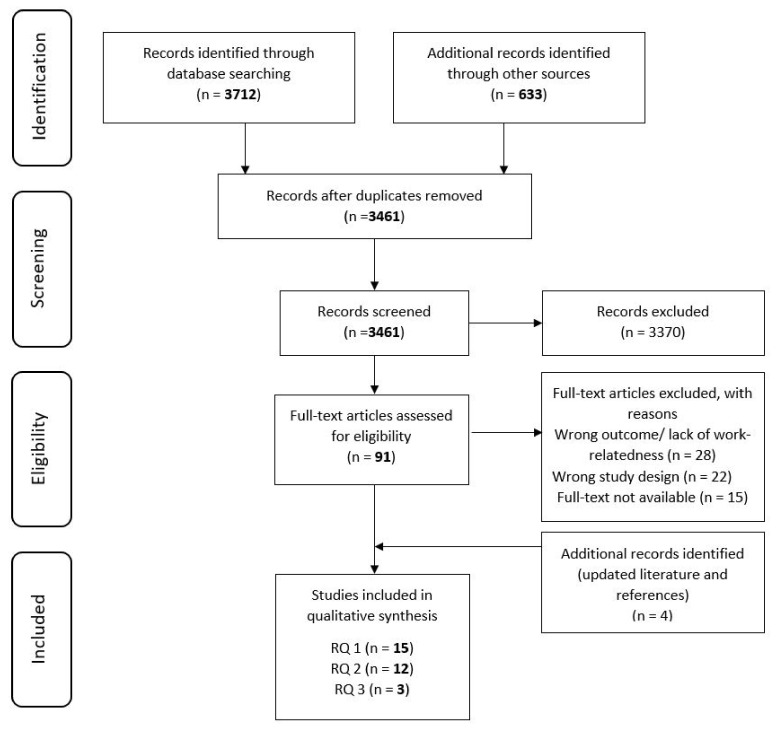
PRISMA flow chart for the study selection process [43].

The literature search resulted in a total of 4345 hits. After removing duplicates, 3461 articles remained for the screening by titles and abstracts. The two reviewers (A.T.E., E.O.) agreed in approximately 95% of the decisions (k = 0.948). The results of the search and the study inclusion process are reported and presented in the flow chart (Figure 1) [43]. After the joint discussion, we included 91 publications for full-text analysis. As part of the full-text analysis, additional articles were excluded or also added (via the snowball system or as newly published articles in the rerun of the search) for the results presented in this scoping review.

We identified a total of 30 articles answering our three research questions (Figure 1) in our search up to mid-January 2021. The included articles were published between 2005 and 2020.

### 3.1. Characteristics of Included Studies

#### Main Review Question (RQ 1)—Conceptualization

Within the scope of answering RQ 1, we first extracted all definitions for “health literacy in the workplace” from the included publications. These definitions are presented in Table 1.

**Table 1:** Definition of health literacy in the workplace as individual competence(s).

Title [Translated German Titles]	Author(s), Year of Publication	Defined Term	Definition *
[Developing health literacies—but how?]	North, Friedrich, and Bernhardt [44], 2010	Health literacies (in the context of nursing personnel)	“Health literacies refer to a person’s abilities and skills to promote, maintain and restore his or her own health; this includes the ability to recognize and evaluate stresses and strains, to develop strategies, to reflect on their effectiveness, and to develop health routines.” (p. 30)
[Safety and health competence through informal learning in the work process]	Hamacher, Eickholt, Lenartz, and Blanco [19], 2012	Work-related safety and health literacy	“Work-related safety and health literacy is the ability and willingness of individuals to make and apply decisions in their daily work that have a positive impact on their health.” (p. 12)
Building a Health Literate Workplace	Wong [45], 2012	Occupational health literacy	“Occupational health literacy is the degree to which workers have the capacity to obtain, communicate, process, and understand occupational health and safety information and services to make appropriate health decisions in the workplace.” (p. 364)
Occupational health literacy and work-related injury among US adolescents	Rauscher and Myers [46], 2014	Occupational health literacy	“OHL [occupational health literacy] is ‘the degree to which individuals have the capacity to obtain, process, and understand basic OSH [occupational safety and health] information and services needed to make appropriate decisions with regard to health and safety at work.’” (p. 81)
[How to promote health competence at work]	Eickholt, Hamacher, and Lenartz [4], 2015	Individual health literacy in the workplace	“Individual health literacy in the occupational context is the ability and willingness to take the initiative in designing one’s personal living and working conditions with regard to health and safety.” (p. 977)
[Memorandum health literacy]	Ernstmann, Bauer, Berens, Bitzer, Bollweg et al. [47], 2020	Health literacy among employees	“Health literacy among employees can be defined as the employee’s ability to make and implement health-promoting decisions in their work and private lives based on evidence-based health knowledge.” (p. e84)

* Translation by ATE in case of German language articles.

These six articles published between 2010 and 2020 define health literacy in the workplace explicitly as an individual competence. Four of the six definitions source from German-language articles and define “work-related health literacy” [19] or “health literacy in the workplace/among employees” [4,44,47], respectively. Wong [45] and Rauscher and Myers [46] define “occupational health literacy”.

Since not all articles define those key terms explicitly, we also extracted and categorized theoretical statements or conceptualizations (nine articles [3,7,48,49,50,51,52,53,54]) in addition to explicit definitions (six articles [4,19,45,46,47]). All articles directly or indirectly address the aspects “access”, “understand”, “assess”, and “apply” that Sørensen et al. [12] synthesized in their conceptualization of health literacy.

Further components of theoretical statements for “health literacy in the workplace” are shown in Table 2 with examples in the footnote of Table 2.

**Table 2:** Patterning chart: main components addressed in theoretical statements on individual work-related health literacy (HL = health literacy).

Term, Author(s), Year of Publication	Explicit Definition	Process That Requires Learning	Individual Prerequisites/Facilitators ^1^	Private Life/Social Context ^2^	Workplace and Work Organization Factors ^3^	System/Environmental Factors ^4^	Work Ability	Employ-Ability	Relation of HL with Company Economic Viability
Individual HL in the workplace Kriegesmann et al. [7], 2005		x ^5^	x	x	x	x	x	x	x
Health literacies North et al. [44], 2010	x	x	x		x		x		x
Work-related safety and HL Hamacher et al. [19], 2012	x	x	x	x	x		x	x	x
HL in the context of rehabilitation and return to work Mårtensson and Hensing [48], 2012		x	x	x	x	x	x		
Occupational HL Wong [45], 2012	x		x		x		x		x
Occupational HL Rauscher and Myers [46], 2014	x	x	x	x	x		x		
Individual HL in the workplace Eickholt et al. [4], 2015	x	x	x	x	x		x		
Individual workplace HL Larsen et al. [49], 2015			x	x	x	x	x		x
Individual HL in the workplace Winter and Seitz [3], 2017		x	x	x	x	x	x	x	x
Work-related HL Georg [50], 2018		x	x	x	x	x	x	x	x
Individual HL in the workplace Gimbel and Lang [51], 2018			x	x	x		x		x
Individual HL in the workplace Uhle and Treier [52], 2019		x	x		x	x	x		
Visual ergonomics literacy Long and Richter [53], 2019			x	x	x		x		
Social insurance literacy Ståhl et al. [54], 2019			x	x	x	x	x	x	
HL among employees Ernstmann et al. [47], 2020	x		x	x	x	x	x		

^1^ Examples for individual prerequisites/facilitators are the ability to act, motivation to act, personal values, and attitudes. ^2^ Examples for private life/social context are being included in small networks, private learning environments, leisure, and exchange with significant others. ^3^ Examples for workplace and work organization factors are working conditions, teamwork, risks, and stresses of work tasks. ^4^ Examples for system/environmental factors are the health care system, social insurances, regulations of the respective state institutions, and professional associations. ^5^ An “x” in the table means that a given component is addressed by the concerning publication.

**Figure 2:**
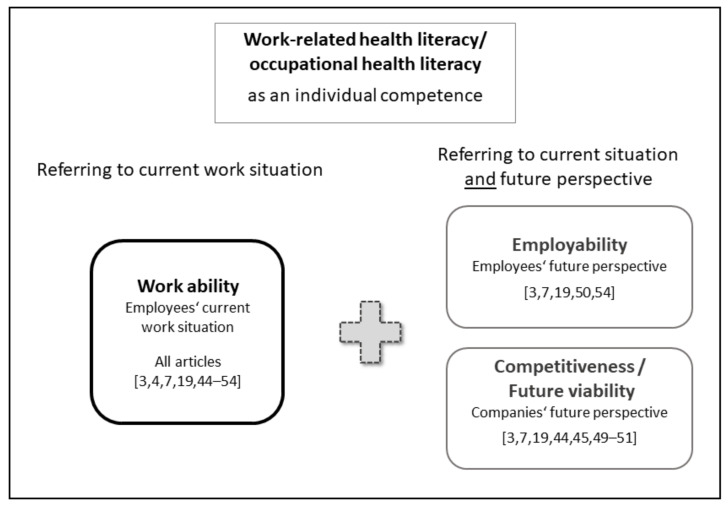
Possible subjects of the concept “individual work-related health literacy” (“occupational health literacy”)—research question 1.

The different approaches to the conceptualization of “health literacy in the workplace” as an individual competence are based on similarities or overlaps of the main components (Table 2 shows which components are addressed in the theoretical articles). Additionally, according to all articles, both individual and work-related factors affect individual work-related health literacy, which in all cases concerns the current work situation. Reference objects of conceptualizations are characteristics of individuals (such as practical use of knowledge and skills or personal values), circumstances of the immediate work environment (such as social relationships in the workplace or the possibility of informal learning at work) as well as aspects of social security (such as the protection or promotion of employee health by governmental or legally prescribed occupational health and safety measures). Another aspect of conceptualizations is the time horizon to which “health literacy in the workplace” refers: all articles refer to the current work situation of employees (work ability) [3,4,7,19,44,45,46,47,48,49,50,51,52,53,54], while some approaches also have a longer-term perspective, on the one hand the entire working life of an employee (employability) [3,7,19,50,54] and/or on the other hand also the future viability (competitiveness) of companies [3,7,19,44,45,49,50,51]. Moreover, we have the works of Long and Richter [53] (visual ergonomics health literacy) as well as Mårtensson and Hensing [48] (health literacy in the context of rehabilitation and return to work) and Ståhl et al. [54] (social insurance health literacy) expressing further possible reference objects of “health literacy in the workplace”. In view of more flexible, mobile workplaces that are increasingly characterized by advances in digitalization and in view of demographic and disease dynamics outlined in the introduction, we included these components in the presentation of individual “work-related” or “occupational health literacy” in Figure 2.

Included literature for RQ 1 dealing with health literacy in the workplace conceptualizes health literacy exclusively as an individual competence of employees and is referred to as “work-related” or “occupational health literacy”. In the following of this text, we refer to this individual health literacy in the workplace as work-related health literacy.

Literature not originating from Germany, such as Wong [45], Rauscher and Myers [46], and Larsen et al. [49], contextualize this individual competence in a larger framework, i.e., a company as a whole organization.

Bringing our working definition of work-related health literacy together with the extracted literature on health literacy in the workplace, the term “occupational health literacy” is to date primarily used outside of Germany. The overview of definitions and theoretical statements shows that all aspects of the conceptualization of health literacy developed by Sørensen et al. (“access”, “understand”, “appraise”, and “apply” health information) [12] appear in some way, even if these aspects were not labeled in exactly the same way: health literacy in the workplace is concerned with knowledge and the application of this knowledge to actions related to safety and health at work. Depending on the research purpose, a definition of individual health literacy in the workplace can either refer to the current work situation only or also include future viability, i.e., employees’ employability or companies’ competitiveness. The relevance of individual work-related health literacy for companies is emphasized by the connection with the long-term viability and competitiveness of companies [3,7,19,44,45,49,50,51].

### 3.2. Interventions for the Promotion of Work-Related Health Literacy (RQ 2)

For RQ 2, in the iterative process of this scoping review, we only included studies on health literacy interventions in the workplace setting for employees as individuals if they provided quantitative results after the implementation of the intervention. Studies reporting only qualitatively on strategies to improve individual work-related health literacy were not included. The work-relatedness of the interventions had to be apparent. Interventions primarily aiming at the workplace, e.g., “mental health first aid”, helping employees recognize health problems in their colleagues (rather than in themselves) were also not included; articles dealing with job-specific safety culture were excluded, too.

Table 3 shows interventions that support work-related health literacy. In view of the variety of outcomes of the interventions and the different types of measurement in different studies (e.g., single self-developed items or (items from) established scales), we report the main outcome and measurement of the interventions. The exact results can be found in the individual studies. The interventions can be categorized into two domains: (a) general work-related health literacy and (b) work-related mental health literacy. There are two studies referring to the same interventions in the same line.

**Table 3:** Interventions for the promotion of individual work-related health literacy with quantitative evaluation results (RQ 2).

Title [Translated German Titles]	Author(s), Year of Publication	Study Design	Domain	Type of Intervention	Population(s) Identified	Setting	Primary Outcome and Measurement	Results
The Effects of a Stress Inoculation Training Program for Civil Servants in Japan: a Pilot Study of a Non-Randomized Controlled Trial	Kawaharada et al., 2009 [55]	Pilot study (non-randomized trial: intervention group and waiting list control group)	Mental health literacy	Stress inoculation training (SIT)	Civil servants (140 civil servants; *n* = 65 intervention group; *n* = 63 waiting list group)	Public organization office Japan	Coping—Ways of Coping Checklist (WCCL; 47 items)	Statistically significant development of problem-solving skills and positive cognition, with a significant effect remaining one month after the intervention
A multifaceted intervention to improve mental health literacy in employees of a multi-campus university: a cluster randomised trial	Reavley et al., 2014 [56]	Cluster randomized trial	Mental health literacy	Whole-of-campus multifaceted intervention	Nine campuses (intervention: 6 clusters, *n* = 162; control: 3 clusters, *n* = 255)	Multi-campus university Australia	Depression—recognition (vignette), anxiety—not named, alcohol use—Alcohol Use Disorders Identification Test (AUDIT; 10 items)	No effects on depression, anxiety levels, and alcohol use but better recognition of depression and greater knowledge
An integrated approach to workplace mental health: an Australian feasibility study	LaMontagne et al., 2014 [57]	Feasibility study	Mental health literacy	Mental health literacy training sessions and job stress intervention	Workers from different worksites (719 workers from 10 worksites/640 workers from 9 worksites)	Different organizations, worksites Australia	Mental health literacy—items developed by beyondblue (27 items)	No significant changes in psychosocial working conditions (job control, job demands, and social support at work), but significant improvements in some aspects of mental health literacy confirmed feasibility of integrating job stress and workplace mental health literacy training
Effective interventions for mental health in male-dominated workplaces	Lee et al., 2014 [58]	Systematic review	Mental health literacy	n.a.	Inclusion of 5 studies	Male-dominated industries, Japan (3 studies) and Finland (2 studies)	e.g., disability, work ability, general health	Effective interventions to address anxiety and depression in male-dominated industries included improving mental health literacy and knowledge, increasing social support, improving access to treatment, providing education for managers, and addressing workload issues
[Improving mental health in the workplace: evaluation of an occupational psychological health promotion program]/[Workplace health promotion for employees with mental disorders]	Latocha, 2015 [59]/Wieland and Latocha, 2015 [60]	Pre-post comparison with intervention and control group	(Mental) health literacy	11 group training sessions	Employees with chronic mental illnesses (intervention group *n* = 34; control group *n* = 41)	Employees in facilities for people with disabilities Germany	Health literacy—German “Gesundheitskompetenz-Fragebogen“ (GKF; 10 items)	Significant improvements for health literacy, functional stress and self-regulation, reduction in anxiety and depressive symptoms
Effects of web-based stress and depression literacy intervention on improving symptoms and knowledge of depression among workers: A randomized controlled trial/ Effects of web-based stress and depression literacy intervention on improving work engagement among workers with low work engagement: An analysis of secondary outcome of a randomized controlled trial	Imamura et al., 2016 [61]/Imamura et al., 2017 [62]	Randomized controlled trial	Mental health literacy	Psychoeducational information website on stress and depression (UTSMed)	Workers with low work engagement (1236 workers at baseline survey (intervention and control group each *n* = 618)/low engagement subgroup intervention *n* = 305 and control group *n* = 318)	Workers Japan	Depressive symptoms—Beck Depression Inventory II (BDI-II; 21 items) (work engagement—short form of the Japanese version of the Utrecht Work Engagement Scale (UWES; 9 items))	Significant intervention effect on improving depressive symptoms was observed at 1-month follow-up only in the high-risk subgroup. Significant effect on work engagement at the 4-month follow-up in the low work engagement subgroup, with a small effect size
GoodYarn: building mental health literacy in New Zealand’s rural workforce	Morgaine et al., 2017 [63]	Pre/post evaluation study	Mental health literacy	Skills-based workshop	Participants in the GoodYarn workshops (*n* = 430)	Rural workforce New Zealand	Mental health literacy—questionnaire at the end of the workshop (14 questions)	Significant positive impact on awareness, confidence in starting a conversation about mental health, and knowledge
Effects of a Classroom Training Program for Promoting Health Literacy Among IT Managers in the Workplace	Fiedler et al., 2019 [64]	Randomized controlled trial	Health literacy	Five-month program for managers	Managers (*n* = 171)	Managers from all management levels and all departments from one IT company Germany	Health literacy—German health literacy questionnaire (29 items)	No significant intervention effect on the primary outcome of general health literacy, psychological well-being and self-rated health significantly decreased, and saliva cortisol levels significantly increased in the second measurement
The effect of strengthening health literacy in nursing homes on employee pain and consequences of pain—a stepped-wedge intervention trial	Larsen et al., 2019 [65]	Stepped-wedge intervention trial	Health literacy	Courses for employees and management and structured dialogs	Employees in nursing homes (*n* = 509)	Six nursing homes Denmark	Musculoskeletal pain intensity—3 questions on pain intensity (e.g., “On a scale from 0–10, what was the highest intensity of pain in your muscles and joints? (0 = no pain, 10 = worst imaginable pain)”)	Feasible and effective in shifting the overall mean pain level downwards
Educational Interventions to Improve Safety and Health Literacy Among Agricultural Workers: A Systematic Review	Coman et al., 2020 [66]	Systematic review	Health literacy	Educational interventions for the improvement of HL and/or safety literacy	Inclusion of 36 studies	Farmers, studies from all over the world	E.g., prevention of farm-induced diseases, accident and injury prevention	Some successful strategies (e.g., lectures, videos, newsletters) with potential to inform public health policies to improve health literacy and develop a safety culture among farmers

The interventions presented here mostly target one aspect of health literacy (e.g., knowledge, understanding health information or coping skills, applying acquired health information), which can be seen in the different approaches and results. We included two review articles. Often, in nine of the 12 studies, interventions aimed to promote mental health literacy. Many of the studies included here and further studies that provide qualitative results make statements about the feasibility of interventions in the workplace. Interventions promoting more general aspects of health literacy may not have been included because they took place in the context of workplace health promotion and only targeted specific aspects such as nutrition and exercise and, thus, were not work-related. The measurement methods and instruments of the intervention studies proved to be very heterogeneous. The overview of specific measurement instruments of work-related health literacy is presented in the results for RQ 3 below.

### 3.3. Measurement Instruments for Work-Related Health Literacy (RQ 3)

Continuing with health literacy as an employee’s individual competence, we present instruments for the measurement of individual work-related health literacy (RQ 3) as result of our literature search. The three self-administered questionnaires were developed and validated within different working populations in the languages Persian [67], English [68], and Thai [69]. Qualitative and quantitative methods were applied for the development and validation of the measurement instruments. Table 4 provides further information on the instruments.

**Table 4:** Development and validation of measurement instruments for individual work-related health literacy (RQ 3).

Instrument	Developer/Author(s), Year of Publication	Measurement	Country of Origin, Language	Study Population	Format/ Number of Items	Factors (or Domains/Dimensions)	Psychometric Properties
Health Literacy Scale for Workers (HELSW)	Azizi et al., 2019 [67]	“Occupational health literacy”	Iran, Persian	*n* = 450 participants (400 men, 50 women)	Self-administered questionnaire with 34 items, 6 factors	Six factors: access, reading, understanding, assessment, decision making, and applying health information, self-efficacy	Exploratory factor analysis: 6 factors with 34 items; the model explained 64.3% of the total variance. Intraclass correlation coefficient and test–retest reliability ranged from 0.72 to 0.84 and 0.69 to 0.86, respectively.
Health Communication Questionnaire (HCQ)	Shannon and Parker, 2020 [68]	“Interactive and critical health literacy within the mining industry”	Australia, English	*n* = 20 mining industry workers; *n* = 20 students in health education; *n* = 3 HL experts; *n* = 46 representative mining industry workers	Self-administered questionnaire with 34 items, 2 factors	Two factors: interactive health literacy and critical health literacy	Demonstrated content validity and face validity; HCQ instrument validity is well supported by results exceeding the target S-CVI/Ave and S-CVI/UA values of 0.90 and 0.80, respectively.
Occupational Health Literacy Scale within the context of Thai working culture (TOHLS-IF)	Suthakorn et al., 2020 [69]	“Occupational health literacy” (informal workers)	Thailand, Thai	*n* = 400 informal workers	Self-administered questionnaire with VAS rating and 38 items, 4 factors	Four factors: ability to gain access, understanding, evaluation, use of occupational health and safety information	Thirty-eight items within 4 factors; model explained 50.2% of the total variance. Confirmatory analysis confirmed satisfactory estimates; high internal consistency and satisfactory reliability (Cronbach’s alpha = 0.98).

HL = health literacy; VAS = visual analog scale

Next to these three studies providing sufficient information on the developed measurement instruments for work-related health literacy according to our definition, there are two studies also reporting on the measurement of occupational health literacy (OHL). Rauscher and Myers [46] surveyed adolescents in telephone interviews about their safety in the workplace with two contributing factors: “OSH [occupational safety and health] information and training” and “OSH [occupational safety and health] knowledge and awareness”. Furthermore, Yusida et al. [70] surveyed occupational health literacy among informal sector workers in Indonesia using 30 items. However, the instruments used in these two studies were not tested for validity and reliability.

## 4. Discussion

The aim of this scoping review was to present the current state of research on health literacy as an employee’s competence. The main research question relates to conceptualizations of health literacy in the workplace. We started from a working definition of work-related health literacy as an individual competence in relation to one’s own work ability and employability. This scoping review offered the opportunity to bundle articles using different country-specific terminologies (e.g., work-related health literacy or occupational health literacy). The literature search revealed different terms for health literacy in the workplace and different approaches that overlap in main components (e.g., the time horizon). The synthesis led us to the result that health literacy in the workplace, as individual competence is about knowledge and its application to actions related to safety and health at work. Depending on the research purpose, a definition (or conceptualization) of individual health literacy in the workplace can either refer to the current work situation only or also include future viability. We continue to refer to this individual competence as work-related heath literacy. Individual work-related health literacy is a resource for employees and is linked to the economic viability of companies [3,7,19,44,45,49,50,51].

Regarding interventions to promote individual work-related health literacy, several interventions were identified that provided quantitative results after being implemented. Examples for these interventions are group trainings, workshops, and educational interventions. In the literature selection process, there were also articles describing (e.g., [71,72,73]) or qualitatively evaluating health literacy interventions in workplaces (e.g., [74])—these insights are important for the implementation of future interventions, but are not covered in this scoping review. Interventions addressing employees’ general health literacy were directed at specific groups of employees: the management level [64], employees in nursing homes [65], or agricultural workers [66]. One intervention targeted employees with mental health problems and addressed partly the general health literacy [60] and partly the mental health literacy of the participating employees [59]. However, most of the interventions included in this scoping review aim at the promotion of mental health literacy. This finding is in line with a recent literature review on the application of the concept “health literacy” in companies [75]. Interventions targeting general health literacy were also less numerous than interventions targeting mental health literacy [75]. In recent years, mental health literacy was the area of most research activities in health literacy research [21]. Mental health literacy refers to the knowledge and beliefs about mental disorders that support their detection, management, or prevention [76]. Mental health literacy is, therefore, often considered in the context of the public or in relation to the corporate workforce; mental health literacy interventions must be developed and applied in a context-specific manner [77]. A well-known example for the promotion of mental health literacy in the workplace are the mental health first aid training courses [78]. Since 2000, the training courses have spread out from Australia and are now available in many countries around the world [78]. These training courses are also offered for the workplace but are not primarily related to individual work-related health literacy, so they are not included in the results section of this scoping review. However, these interventions can also have a positive effect on one’s own mental health literacy, as Kitchener and Jorm identified [79]. In some cases, mental health literacy interventions showed significant effects on the primary outcome [55,59,61,63] or improvements in other outcomes [56,57,62]. However, all interventions contributed to raise awareness of mental health in the workplace and to changes in attitudes toward mental health [56,57,61,62,63]. It was found generally that interventions increasing one’s own abilities should be more focused on the work or the working conditions and that health literacy can be improved through targeted interventions [55,60,75]. Other interventions to promote health literacy in the workplace are interventions that are directed at specific employees (e.g., managers), but whose effects primarily affect the workplace (i.e., structural preventive measures) rather than employees themselves. An example that illustrates this aspect is a training for managers helping to support the mental health needs of their employees (but not their own) [80]. Lack of work-relatedness was a frequent exclusion criterion (*n* = 28). Interventions to promote health literacy in general but only using the workplace setting (e.g., workplace health promotion offers) were excluded because the immediate objective of the activities was not to maintain (or restore or improve) work ability. In our literature search, the term “health literacy” was required in the title or abstract; otherwise, articles were not included, which may have resulted in some articles with relevant content not being considered in this scoping review.

Interestingly, in none of the intervention studies reported (see Table 3) a comprehensive instrument to measure work-related health literacy was applied. Beyond the validation studies described in Table 4, we found two publications reporting on measurement instruments for measuring individual work-related health literacy or occupational health literacy. To date, there have been no publications evaluating interventions using any of these instruments. The various measures of outcomes in intervention studies promoting individual work-related or occupational health literacy (Table 3), and the measurement instruments available to date (Table 4) indicate a need for validated measurement instruments designed for the workplace context. Because no specific measurement instrument was available, in an intervention study among casting factory workers, general health literacy was measured and linked to workers’ perceptions of occupational health and safety training [14]. Suthakorn et al. [69] recommend the development of occupational health literacy measurement instruments tailored to specific work settings in different cultures.

In addition to the instruments presented in Table 4, there are two other measurement instruments, including a German one [81] translated from the original English version [82]. These instruments assess only the knowledge aspect of mental health literacy in respect of one’s colleagues at work and, for this reason, were not included as measurement instruments in this review. Furthermore, there is an instrument that asks educators about their knowledge regarding the mental health of their students [83]. This shows that “mental health literacy” as a defined term has received attention and finds application in the context of employment.

For future instruments measuring individual work-related or occupational health literacy, we recommend assessing all aspects of Sørensen et al.’s conceptualization [12]. Since more flexible career paths may be possible nowadays, we recommend also including relevant aspects of new forms of work to cover the individual aspect of employability into the development of instruments.

The relevance and the advantage of the issue “health literacy in the workplace” may be found in relation to peoples’ changing work environments and employment biographies. [25,26,27] The COVID-19 pandemic imposes far-reaching impacts on societies, health care systems, workplaces, and individuals [84]. Changes in the nature of work, the use of technologies at work, business structures, status of employment, hierarchies as well as relationships at work as outlined in a report of the European Agency for Safety and Health at Work [2] are accelerating due to the COVID-19 pandemic and also affect workplaces where consequences of increasing digitalization had previously been slower [85].

As described in the background, health literacy can be valuable in promoting work ability. Work ability is the basis for employability and employment [17] and is becoming more important in view of changes in working and living conditions. Living conditions are also affected, as constant accessibility increases and the boundaries between work and private life can become increasingly blurred [2]. Interventions that are tailored to the needs of employees in companies may have a more successful implementation because they are close to the company, the sense of responsibility is given, and an added value can be seen for both employees and employers. For this reason, interventions to promote individual work-related health literacy might be better accepted than offers (e.g., workplace health promotion) to promote general health literacy in the company.

### 4.1. Limitations of the Study

We aimed to identify all relevant articles on employee health literacy. However, there were more measures and interventions described or in planning not only for employees themselves. Articles on organizational health literacy—in which health literacy is conceived of an attribute belonging to organizations or companies (and not to working individuals) [86]—were not included because we started from an individual characteristic with our working definition and the outcome “work ability” or “individual employability”; we wanted to address employees with their abilities to stay safe and healthy during their working life. We narrowed the inclusion criteria for articles on interventions. For example, our scoping review does not address what experience has been gained in implementing interventions to promote work-related or occupational health literacy. There may be quantitative intervention studies that promote one aspect of health literacy without mentioning “health literacy” in the title/abstract; these articles were not retrieved via the literature search. Another limitation of this scoping review relates to the inclusion criteria. Thus, articles that focus on general health literacy and articles that focus on work–life balance were excluded. Articles on these areas may have an indirect impact on the individual work-related health literacy and the maintenance of work ability or employability. In particular, articles on work–life balance are of special importance in a pandemic situation such as the current COVID-19 crisis with increased offers of mobile working. Additionally, emerging requirements regarding work-related health literacy, such as those resulting from the pandemic, have not yet been considered in this scoping review.

Only articles in German and English were included, leaving some activities from other language countries out of the scope of this review. In addition, due to the breadth of the research questions, relevant articles on a particular research question may not have been found and may not have received attention in this review.

### 4.2. Future Research

In this scoping review, we focused on an occupational work environment with employees working within an organization. Mobile work and forms of more flexible work including self-employed or platform work [87] require a more detailed consideration.

We can confirm that there is a lack of reliable and valid measurement instruments for work-related or occupational health literacy. To date, there is no standardized measurement instrument for individual health literacy that has been designed specifically for workplaces in general.

## 5. Conclusions and Recommendations

The strength of individual work-related or occupational health literacy is its direct relation to one’s own work ability and employability. Workplaces, where most people spend a significant proportion of their lives, can be advantages in terms of acceptance and implementation of measures. It can be suspected that the degree of acceptance of measures to increase work-related health literacy will be higher than the acceptance of measures to promote general health literacy in the workplace setting.

Considering the components of individual work-related health literacy and the country- or environment-specific characteristics of workplaces, the applicability of the conceptualization of work-related health literacy from other countries and different workplaces is not without obstacles. Social security and health care systems as well as labor market characteristics vary between countries. Thus, we recommend the development of country-specific instruments for the assessment of individual work-related health literacy and to measure both mental and physical work-related health literacy. In our view, a recommendation for future research is also to consider precarious working conditions as one aspect of working conditions.

The occupational safety and health of the future should also focus more on strengthening health literacy, but at the same time, should not consider itself released from the task of structural prevention [66], e.g., by fostering occupational safety culture [38]. Especially in awareness of the continuing shortages of skilled workers, demographic developments, and new, changed working conditions, individual health literacy is of growing importance. We encourage the development of targeted interventions with the aim of maintaining and promoting work ability as well as participation in employment.

## Supplementary Materials

The following are available online at https://www.mdpi.com/article/10.3390/ijerph18199945/s1, Table S1: Preferred reporting items for systematic reviews and meta-analyses extension for scoping reviews (PRISMA-ScR) checklist [30].

## Appendix A

Search strategy for PubMed and adapted search strategies.

((work [MeSH Terms]) OR (workplace [MeSH Terms]) OR (employment [MeSH Terms:noExp]) OR (occupations [MeSH Terms]) OR (occupational groups [MeSH Terms]) OR (occupational medicine [MeSH Terms]) OR (occupational health services [MeSH Terms]) OR (“Occupational Diseases”[Mesh Terms:noExp]) OR (occupation*[Title/Abstract]) OR (“occupational health”[Title/Abstract]) OR (“occupational medicine”[Title/Abstract]) OR (work-related [Title/Abstract]) OR (“working environment*” [Title/Abstract]) OR (workplace*[Title/Abstract]) OR (“work place”[Title/Abstract]) OR (“work site”[Title/Abstract]) OR (Worksite* [Title/Abstract]) OR (Workplace[Title/Abstract]) OR (“at work” [Title/Abstract]) OR (job [Title/Abstract]) OR (workstation [Title/Abstract]) OR (employment [Title/Abstract]) OR (employability [Title/Abstract]) OR (Employee* [Title/Abstract]) OR (Personnel [Title/Abstract]) OR (Worker* [Title/Abstract]))
((health literacy [MeSH Terms]) OR (“health literacy” [Title/Abstract]) OR (“health literacies” [Title/Abstract]) OR (“health competence” [Title/Abstract]) OR (“health illiteracy” [Title/Abstract]))
participants/context AND concept

CINAHL (interface: EBSCOhost)
MH “Work+” OR MH “Work Environment+” OR MH “Employment” OR MH “Named Groups by Occupation+” OR MH “Occupational Medicine” OR MH “Occupational Health Services+” OR MH “Occupational Diseases” OR AB occupation* OR TI occupation* OR AB “occupational health” OR TI “occupational health” OR AB “occupational medicine” OR TI “occupational medicine” OR AB work-related OR TI work-related OR AB “working environment” OR TI “working environment” OR AB workplace* OR TI workplace* OR AB “work-site” OR TI “work-site” OR AB Worksite* OR TI Worksite* OR AB “at work” OR TI “at work” OR AB job OR TI job OR AB workstation OR TI workstation OR AB employment OR TI employment OR AB employability OR TI employability OR AB Employee* OR TI Employee* OR AB Personnel OR TI Personnel OR AB Worker* OR TI Worker* OR AB (working N1 environment*) OR TI (working N1 environment*)
MH “Health Literacy” OR AB “health literacy” OR TI “health literacy” OR AB “health literacies” OR TI “health literacies” OR AB “health competence” OR TI “health competence” OR AB “health illiteracy” OR TI “health illiteracy”
participants/context AND concept

APA PsycInfo (interface: EBSCOhost)
TI occupation* OR AB occupation* OR TI “occupational health” OR AB “occupational health” OR TI “occupational medicine” OR AB “occupational medicine” OR TI work-related OR AB work-related OR TI “working environment” OR AB “working environment” OR TI workplace* OR AB workplace* OR TI “work place” OR AB “work place” OR TI “work site” OR AB “work site” OR TI worksite* OR AB worksite* OR TI workplace OR AB workplace OR TI “at work” OR AB “at work” OR TI job OR AB job OR TI workstation OR AB workstation OR TI employment OR AB employment OR TI employability OR AB employability OR TI employee* OR AB employee* OR TI personnel OR AB personnel OR TI worker* OR AB worker* OR TI “working environment*” OR AB “working environment*” OR ((((DE “Work (Attitudes Toward)”) OR (DE “Personnel”)) OR (DE “Occupational Health”)) OR (DE “Occupations”)) OR (DE “Employability”)
TI “health literacy” OR AB “health literacy” OR TI “health literacies” OR AB “health literacies” OR TI “health competence” OR AB “health competence” OR TI “health illiteracy” OR AB “health illiteracy” OR (DE “Health Literacy”)
participants/context AND concept

PSYNDEX Literature with PSYNDEX Tests (interface: EBSCOhost)
TI occupation* OR AB occupation* OR TI “occupational health” OR AB “occupational health” OR TI “occupational medicine” OR AB “occupational medicine” OR TI work-related OR AB work-related OR TI “working environment” OR AB “working environment” OR TI workplace* OR AB workplace* OR TI “work place” OR AB “work place” OR TI “work site” OR AB “work site” OR TI worksite* OR AB worksite* OR TI workplace OR AB workplace OR TI “at work” OR AB “at work” OR TI job OR AB job OR TI workstation OR AB workstation OR TI employment OR AB employment OR TI employability OR AB employability OR TI employee* OR AB employee* OR TI personnel OR AB personnel OR TI worker* OR AB worker* OR TI “working environment*” OR AB “working environment*” OR ((((DE “Work (Attitudes Toward)”) OR (DE “Personnel”)) OR (DE “Occupational Health”)) OR (DE “Occupations”)) OR (DE “Employability”)
TI “health literacy” OR AB “health literacy” OR TI “health literacies” OR AB “health literacies” OR TI “health competence” OR AB “health competence” OR TI “health illiteracy” OR AB “health illiteracy” OR (DE “Health Literacy”)
participants/context AND concept

Search strategy for additional hand search (For all sources except SpringerLink: Two independent searches for the German and English term were performed).

The Joint German Occupational Safety and Health Strategy (Gemeinsame Deutsche Arbeitsschutzstrategie, GDA) - Gesundheitskompetenz - “health literacy”
Federal Institute for Occupational Safety and Health (Bundesanstalt für Arbeitsschutz und Arbeitsmedizin, BAuA) - Gesundheitskompetenz - “health literacy”
Occupational medicine, social medicine, environmental medicine, Journal for medical prevention (Arbeitsmedizin, Sozialmedizin, Umweltmedizin, Zeitschrift für medizinische Prävention, ASU) - Gesundheitskompetenz - “health literacy”
Zentralblatt für Arbeitsmedizin, Arbeitsschutz und Ergonomie (via SpringerLink) - Gesundheitskompetenz - “health literacy”
Zeitschrift für Arbeitswissenschaft (via SpringerLink) - Gesundheitskompetenz - “health literacy”
SpringerLink (Gesundheitskompetenz (Arbeitsmedizin OR Arbeitsplatz OR Arbeitsfähigkeit OR Erwerbsteilhabe)) OR (“health literacy“ (Arbeitsmedizin OR Arbeitsplatz OR Arbeitsfähigkeit OR Erwerbsteilhabe)) Filter: German
